# Supplementary material for: Serum and Urinary Matrix Metalloproteinase-9 Concentrations in Dehydrated Horses
Source: Animals (Basel). 2023 Dec 7;13(24):3776. doi: 10.3390/ani13243776 (PMC10741069; doi:10.3390/ani13243776)
Supplement: Supplementary file 1 [file animals-13-03776-s001.zip › Supplementary Table S2_urin.pdf]

Table S2. Urine MMP-9 measurements of 40 dehydrated horses and 4 healthy horses including mean, variation, standard deviation, and standard error of the mean of all six measurements of each sample at timepoints 0h, 12, 24h, 48h.

Horse number	Time point	mean [ng/ml]	variation	standard deviation	standard error of the mean
**1**	0h	692.0	4039.2	63.6	44.9
	12h	NA	NA	NA	NA
	24h	NA	NA	NA	NA
	48h	NA	NA	NA	NA
**2**	0h	277.4	960.6	31.0	21.9
	12h	NA	NA	NA	NA
	24h	NA	NA	NA	NA
	48h	NA	NA	NA	NA
**3**	0h	817.5	3555.9	59.6	42.2
	12h	422.7	2003.6	44.8	31.7
	24h	359.4	3666.4	60.6	42.8
	48h	NA	NA	NA	NA
**4**	0h	804.0	2663.1	51.6	36.5
	12h	198.5	383.7	19.6	13.9
	24h	404.4	5290.2	72.7	51.4
	48h	433.3	575.1	24.0	17.0
**5**	0h	360.2	1162.2	34.1	24.1
	12h	221.9	297.3	17.2	12.2
	24h	202.1	699.8	26.5	18.7
	48h	198.7	18.2	4.3	3.0
**6**	0h	331.3	2071.0	45.5	32.2
	12h	419.1	1274.7	35.7	25.2
	24h	585.8	1411.2	37.6	26.6
	48h	246.0	132.5	11.5	8.1
**7**	0h	445.3	7689.3	87.7	62.0
	12h	132.0	22.8	4.8	3.4
	24h	260.0	840.0	29.0	20.5
	48h	480.4	10564.6	102.8	72.7
**8**	0h	572.0	2898.9	53.8	38.1
	12h	NA	NA	NA	NA
	24h	NA	NA	NA	NA
	48h	NA	NA	NA	NA
**9**	0h	592.7	2091.0	45.7	32.3
	12h	310.8	119.0	10.9	7.7
	24h	NA	NA	NA	NA
	48h	NA	NA	NA	NA
**10**	0h	913.3	10465.5	102.3	72.3
	12h	339.6	1417.2	37.6	26.6
	24h	408.8	4203.4	64.8	45.8
	48h	427.5	990.7	31.5	22.3
**11**	0h	269.4	1016.2	31.9	22.5

	12h	NA	NA	NA	NA
	24h	NA	NA	NA	NA
	48h	NA	NA	NA	NA
**12**	0h	660.3	2968.7	54.5	38.5
	12h	NA	NA	NA	NA
	24h	NA	NA	NA	NA
	48h	NA	NA	NA	NA
**13**	0h	216.2	262.0	16.2	11.4
	12h	205.8	365.0	19.1	13.5
	24h	162.4	592.8	24.3	17.2
	48h	155.9	40.7	6.4	4.5
**14**	0h	172.0	573.3	23.9	16.9
	12h	188.5	282.2	16.8	11.9
	24h	200.6	1560.8	39.5	27.9
	48h	270.4	576.1	24.0	17.0
**15**	0h	477.1	3893.7	62.4	44.1
	12h	224.0	488.2	22.1	15.6
	24h	298.7	947.6	30.8	21.8
	48h	597.0	8379.9	91.5	64.7
**16**	0h	571.1	1467.6	38.3	27.1
	12h	NA	NA	NA	NA
	24h	NA	NA	NA	NA
	48h	NA	NA	NA	NA
**17**	0h	NA	NA	NA	NA
	12h	437.5	3902.2	62.5	44.2
	24h	295.1	841.1	29.0	20.5
	48h	130.1	16.3	4.0	2.9
**18**	0h	477.9	5282.4	72.7	51.4
	12h	211.4	279.9	16.7	11.8
	24h	351.3	2427.3	49.3	34.8
	48h	246.9	269.7	16.4	11.6
**19**	0h	484.8	3761.9	61.3	43.4
	12h	339.7	1521.3	39.0	27.6
	24h	462.2	2840.6	53.3	37.7
	48h	411.3	451.2	21.2	15.0
**20**	0h	529.8	5515.5	74.3	52.5
	12h	NA	NA	NA	NA
	24h	NA	NA	NA	NA
	48h	NA	NA	NA	NA
**21**	0h	486.7	2395.8	48.9	34.6
	12h	301.1	411.7	20.3	14.3
	24h	332.0	1260.3	35.5	25.1
	48h	137.5	19.4	4.4	3.1
**22**	0h	263.8	1836.7	42.9	30.3
	12h	401.6	3597.2	60.0	42.4
	24h	249.2	200.4	14.2	10.0
	48h	299.0	408.7	20.2	14.3
**23**	0h	762.1	933.8	30.6	21.6
	12h	464.6	7051.4	84.0	59.4

	24h	NA	NA	NA	NA
	48h	NA	NA	NA	NA
**24**	0h	587.1	1930.4	43.9	31.1
	12h	294.9	2135.0	46.2	32.7
	24h	194.5	1382.8	37.2	26.3
	48h	258.3	64.8	8.0	5.7
**25**	0h	393.0	1343.8	36.7	25.9
	12h	404.7	4219.9	65.0	45.9
	24h	307.7	2686.7	51.8	36.7
	48h	242.8	198.4	14.1	10.0
**26**	0h	1083.2	10723.0	103.6	73.2
	12h	NA	NA	NA	NA
	24h	NA	NA	NA	NA
	48h	NA	NA	NA	NA
**27**	0h	559.7	3560.2	59.7	42.2
	12h	160.0	422.6	20.6	14.5
	24h	226.1	1279.4	35.8	25.3
	48h	153.7	167.6	12.9	9.2
**28**	0h	895.6	4470.6	66.9	47.3
	12h	415.9	3701.7	60.8	43.0
	24h	228.6	580.4	24.1	17.0
	48h	247.2	402.2	20.1	14.2
**29**	0h	822.3	9326.0	96.6	68.3
	12h	394.9	2210.7	47.0	33.2
	24h	475.1	7966.7	89.3	63.1
	48h	NA	NA	NA	NA
**30**	0h	383.1	4422.0	66.5	47.0
	12h	277.2	550.4	23.5	16.6
	24h	224.2	769.4	27.7	19.6
	48h	703.0	910.8	30.2	21.3
**31**	0h	1300.2	79444.9	281.9	199.3
	12h	1139.4	21091.2	145.2	102.7
	24h	889.2	2331.6	48.3	34.1
	48h	328.7	62.7	7.9	5.6
**32**	0h	865.9	4342.9	65.9	46.6
	12h	1213.8	46489.4	215.6	152.5
	24h	NA	NA	NA	NA
	48h	NA	NA	NA	NA
**33**	0h	1715.4	474292.2	688.7	487.0
	12h	NA	NA	NA	NA
	24h	NA	NA	NA	NA
	48h	NA	NA	NA	NA
**34**	0h	1384.9	82281.7	286.8	202.8
	12h	539.7	7122.8	84.4	59.7
	24h	1048.3	16510.0	128.5	90.9
	48h	744.2	1955.2	44.2	31.3
**35**	0h	2690.5	519430.5	720.7	509.6
	12h	NA	NA	NA	NA
	24h	NA	NA	NA	NA

	48h	NA	NA	NA	NA
**36**	0h	3597.2	1176857.0	1084.8	767.1
	12h	750.2	13663.0	116.9	82.7
	24h	406.0	4846.9	69.6	49.2
	48h	NA	NA	NA	NA
**37**	0h	723.9	9544.1	97.7	69.1
	12h	184.2	576.8	24.0	17.0
	24h	181.4	306.3	17.5	12.4
	48h	174.3	106.9	10.3	7.3
**38**	0h	1100.3	97053.5	311.5	220.3
	12h	355.6	3193.9	56.5	40.0
	24h	907.9	17788.8	133.4	94.3
	48h	NA	NA	NA	NA
**39**	0h	693.6	1946.7	44.1	31.2
	12h	NA	NA	NA	NA
	24h	NA	NA	NA	NA
	48h	NA	NA	NA	NA
**40**	0h	NA	NA	NA	NA
	12h	NA	NA	NA	NA
	24h	NA	NA	NA	NA
	48h	NA	NA	NA	NA
**Healthy 1**	0h	1.2	0.1	0.26	0.11
	12h	NA	NA	NA	NA
	24h	NA	NA	NA	NA
	48h	NA	NA	NA	NA
**Healthy 2**	0h	0.2	0.0	0.07	0.03
	12h	NA	NA	NA	NA
	24h	NA	NA	NA	NA
	48h	NA	NA	NA	NA
**Healthy 3**	0h	0.6	0.0	0.05	0.02
	12h	NA	NA	NA	NA
	24h	NA	NA	NA	NA
	48h	NA	NA	NA	NA
**Healthy 4**	0h	0.6	0.0	0.17	0.06
	12h	NA	NA	NA	NA
	24h	NA	NA	NA	NA
	48h	NA	NA	NA	NA
